# The Use of P63 Immunohistochemistry for the Identification of Squamous Cell Carcinoma of the Lung

**DOI:** 10.1371/journal.pone.0012209

**Published:** 2010-08-17

**Authors:** Esther Conde, Bárbara Angulo, Pilar Redondo, Oscar Toldos, Elena García-García, Ana Suárez-Gauthier, Belén Rubio-Viqueira, Carmen Marrón, Ricardo García-Luján, Montse Sánchez-Céspedes, Angel López-Encuentra, Luis Paz-Ares, Fernando López-Ríos

**Affiliations:** 1 Laboratorio de Dianas Terapéuticas, Centro Integral Oncológico “Clara Campal”, Hospital Universitario Madrid Sanchinarro, Universidad San Pablo-CEU, Madrid, Spain; 2 Pathology, Thoracic Surgery and Hospital Universitario 12 de Octubre, Madrid, Spain; 3 Oncology Department, Hospital Universitario Madrid Sanchinarro, Universidad San Pablo-CEU, Madrid, Spain; 4 Thoracic Surgery, Hospital Universitario 12 de Octubre, Madrid, Spain; 5 Pulmonary Department, Hospital Universitario 12 de Octubre, Madrid, Spain; 6 Genes and Cancer Group, Programa de Epigenetica y Biologia del Cancer-PEBC, Institut d'Investigacions Biomediques Bellvitge (IDIBELL), L'Hopitalet de Llobregat, Barcelona, Spain; 7 Oncology Department, Instituto de Biomedicina de Sevilla (IBIS) and Hospital Universitario Virgen del Rocío, Sevilla, Spain; Health Canada, Canada

## Abstract

**Introduction:**

While some targeted agents should not be used in squamous cell carcinomas (SCCs), other agents might preferably target SCCs. In a previous microarray study, one of the top differentially expressed genes between adenocarcinomas (ACs) and SCCs is *P63*. It is a well-known marker of squamous differentiation, but surprisingly, its expression is not widely used for this purpose. Our goals in this study were (1) to further confirm our microarray data, (2) to analize the value of P63 immunohistochemistry (IHC) in reducing the number of large cell carcinoma (LCC) diagnoses in surgical specimens, and (3) to investigate the potential of P63 IHC to minimize the proportion of “carcinoma NOS (not otherwise specified)” in a prospective series of small tumor samples.

**Methods:**

With these goals in mind, we studied (1) a tissue-microarray comprising 33 ACs and 99 SCCs on which we performed P63 IHC, (2) a series of 20 surgically resected LCCs studied for P63 and TTF-1 IHC, and (3) a prospective cohort of 66 small thoracic samples, including 32 carcinoma NOS, that were further classified by the result of P63 and TTF-1 IHC.

**Results:**

The results in the three independent cohorts were as follows: (1) P63 IHC was differentially expressed in SCCs when compared to ACs (p<0.0001); (2) half of the 20 (50%) LCCs were positive for P63 and were reclassified as SCCs; and (3) all P63 positive cases (34%) were diagnosed as SCCs.

**Conclusions:**

P63 IHC is useful for the identification of lung SCCs.

## Introduction

**Figure 1:**
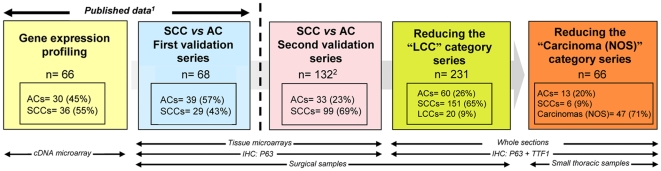
Summary of the methodology used in the different series. 1. See reference 3. 2. This series also included 10 large cell carcinomas and 4 sarcomatoid carcinomas.

The arrival, approximately a decade ago, of global gene expression profiling studies meant an improvement in the classification of many malignant neoplasias [1]. However, the practical impact on lung carcinoma classification has been comparatively small [2]. In a previous microarray study, we compared primary lung adenocarcinoma (AC) with squamous cell carcinoma (SCC) in order to find new immunohistochemical antibodies that could improve the accuracy of the distinction in daily practice [3]. Our approach was very robust because cases included in the analysis were surgical specimens re-classified by two thoracic pathologists (EC and FL-R) according to the 2004 WHO Classification [4]. One of the top differentially expressed genes that we found was *P63*, a well-known marker of squamous differentiation but, surprisingly, its expression is not widely used for this purpose in pathology laboratories worldwide. Indeed, this result was validated with a tissue microarray (TMA) (Fig. 1 and Table 1).

**Table 1:** Validation of P63 IHC as a marker of squamous differentiation.

	1° IHC validation series#		2° IHC validation series	
	SCC (n = 29)	AC (n = 39)	SCC (n = 91*)	AC (n = 29†)
P63 negative	7 (24%)	29 (74%)	42 (46%)	27 (93%)
P63 positive	22 (76%)	10 (26%)	49 (54%)	2 (7%)
	P<0.001		P<0.001	

# Published data (see reference 3).

*8 SCCs were not available for immunostaining evaluation.

† 4 ACs were not available for immunostaining evaluation.

Nowadays we are facing a situation in which some new targeted agents should not be used in SCCs, not only because they do not provide better response rates (pemetrexed), but also because their use in this histological type is associated with life-threatening complications (i.e. bevacizumab) [5]–[7]. To further complicate the field, other agents (i.e., anti-IGFR) might only (or preferably) increase the response rate of SCC [8].

Given the recent need to identify lung SCCs, we tried to further confirm our previous findings in another independent series. At the same time, we sought to investigate the feasibility of this approach to reduce the “large cell carcinoma (LCC)” category in surgical specimens and to increase the number of specific diagnoses in a prospective series of small thoracic samples.

## Methods

### Ethics Statement

Written informed consent was obtained from all participants involved. We obtained ethics approval from the ethics committees at all institutions where samples were analyzed.

### Tumor samples and histological characteristics

Small cell lung carcinomas were excluded from the study. Fig. 1 summarizes our methodology, including our published data [3]. To further confirm our P63 microarray data in another independent cohort (second validation series), we started studying 146 patients who underwent resection of staged pI-II NSCLCs at “12 de Octubre” University Hospital between 1993 and 1997. Pathological characteristics of the tumors included in the analysis were as follows: 33 (23%) ACs; 99 (69%) SCCs; 10 (7%) LCCs and four (3%) sarcomatoid carcinomas (SCs). This study was performed on TMAs and only P63 IHC was performed. Next, our aim was to investigate the utility of P63 and also TTF-1 immunostaining to reduce the number of LCC diagnoses on surgically resected lung specimens. We included 231 patients (reducing the “LCC” category series) who underwent resection of staged pI-II NSCLCs at “12 de Octubre” University Hospital between 1997 and 2003. Pathological characteristics of the tumors included were as follows: 60 (26%) ACs; 151 (65%) SCCs; and twenty (9%) LCCs. The study was performed on whole tissue sections. Afterwards, we investigated the feasibility of the same approach in a prospective cohort (reducing the “carcinoma NOS” category series) of 66 small thoracic samples (51 bronchoscopic biopsies and fifteen core-needle biopsies) from the Targeted Therapies Laboratory at the Madrid Sanchinarro University Hospital. The classification of the tumors was as follows: 47 (71%) carcinoma not otherwise specified (NOS); 13 (20%) ACs; and six (9%) SCCs. Thirty two of the 47 undefined carcinomas (27 bronchoscopic biopsies and five core-needle biopsies) could be further studied for P63 and TTF-1. In the remaining cases in this group, all tissue had been previously used for mutation analysis (data not shown). After clinical evaluation, all but two cases were considered unresectable. In spite of not having the “gold standard” of surgical excision, we chose to study this cohort because it is precisely in patients with advanced lung carcinoma in which our approach would be most helpful.

### Immunohistochemistry

We performed immunohistochemical (IHC) staining of P63 (4A4, 1:50 dilution; DAKO) in all cohorts. The anti-P63 monoclonal antibody 4A4 recognizes all 6 isoforms (total P63 expression): TAp63α, TAp63β, TAp63γ, ΔNp63α, ΔNp63β, ΔNp63γ [9]. IHC staining of TTF-1 (8G7G3/1, 1:200; DAKO) was also carried out in the last two series. After incubation, immunodetection was done with the DAKO EnVision Visualization Method (Dako, Glostrup, Denmark), with diaminobenzidine chromogen as the substrate. Sections were counterstained with hematoxylin. Immunostaining was evaluated by two different pathologists (EC and FL-R), using criteria based on published cut-offs, as follows. P63: scored positive when high intensity staining was present on ≥50% of tumor cells; the remainder was scored negative [10]. TTF-1: scored positive when staining was present on ≥5% of tumor cells; the remainder was scored negative [11]. For both antibodies, only distinct and intense nuclear staining was considered positive. For all LCCs with neuroendocrine morphology, immunostaining for CD56 (123C3, 1:50 dilution; DAKO) and synaptophysin (SY38, 1:25 dilution; DAKO) also was performed to confirm neuroendocrine differentiation.

### Statistical analysis

Frequencies were compared either by Fisher's exact test or by the X^2^ contingency test. Differences of p<0.05 were considered statistically significant. Analyses were performed using the SPSS program, version 10.0.5 (SPSS Inc, Chicago, IL).

## Results

### Validation of P63 immunohistochemical expression as a marker of squamous differentiation

Results of P63 expression are summarized in Table 1. In the first validation series, sensitivity = 0.76, specificity = 0.74, positive predictive value = 0.69, negative predictive value = 0.81 and accuracy = 0.75. In the second validation series, two of 29 ACs (7%) compared with 49 of 91 SCCs (54%) were positive for P63 IHC (p<0.001). Sensitivity, specificity, positive predictive value, negative predictive value and accuracy were 0.54, 0.93, 0.96, 0.39 and 0.63, respectively.

### Value of P63 and TTF-1 immunohistochemistry in reducing the “large cell carcinoma” category in surgical specimens

**Figure 2:**
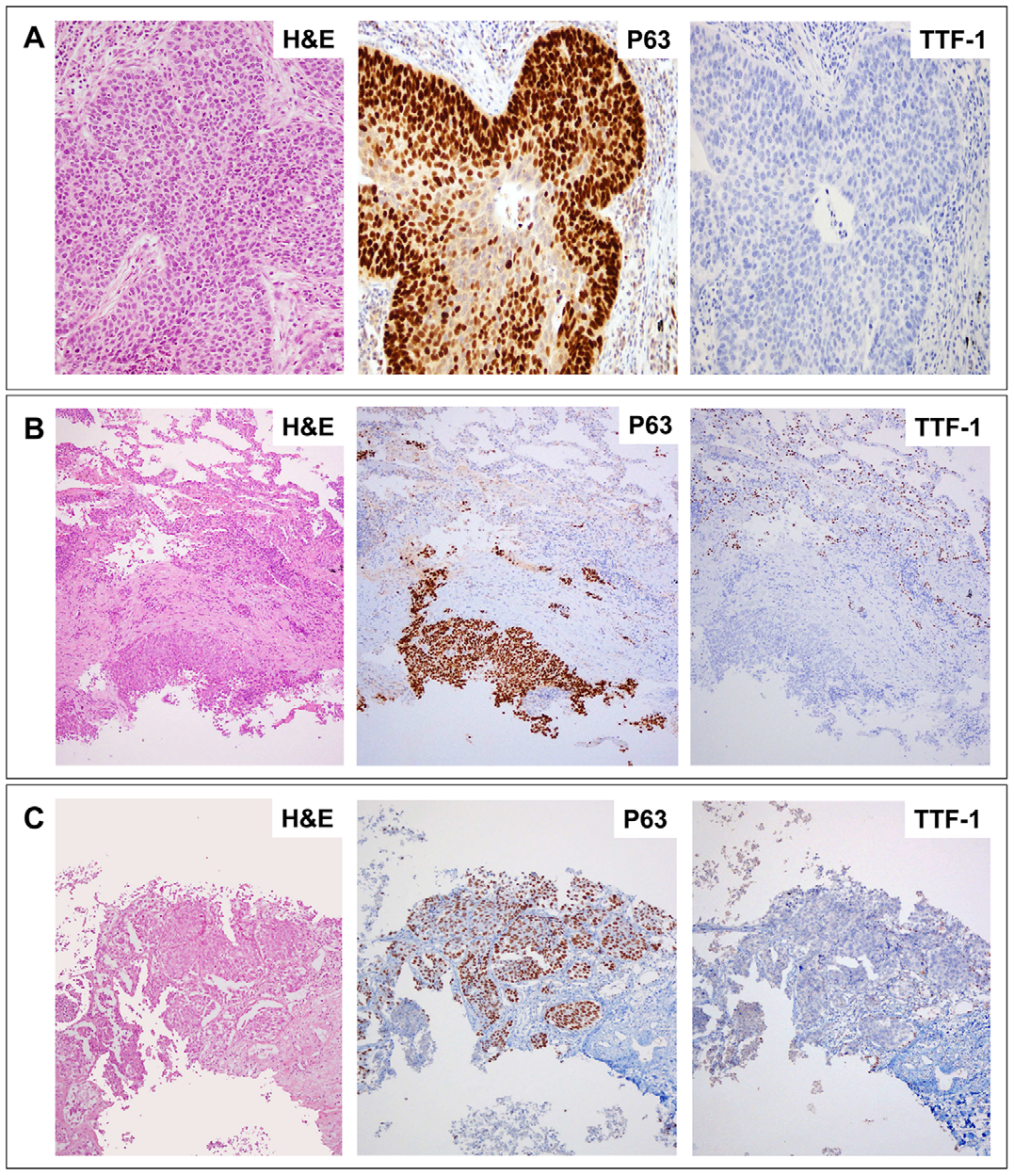
P63 and TTF-1 immunohistochemistry. Cases of LCC (A), carcinoma NOS on bronchoscopic biopsy (B) and carcinoma NOS on core-needle biopsy (C) are shown. They were all re-classified as SCCs, showing a mutually exclusive pattern: P63 positive and TTF-1 negative. For both antibodies only distinct nuclear staining was considered positive. High-intensity staining in ≥50% of tumor cells was scored as positive for P63.

On the basis of our previous results of P63 IHC as a squamous marker and the published data demonstrating that TTF-1 is essentially not detected in SCCs, we assessed the utility of both antibodies for re-classifying 20 LCCs (Table 2) [12], [13]. Half of the 20 (50%) LCCs were positive for P63 and were re-classified as SCCs. All but two P63 positive cases did not express TTF-1 (Fig. 2A). The remaining eight cases were positive for TTF-1 and seven were considered ACs. Finally, three carcinomas exhibited features of neuroendocrine differentiation (palisading, necrosis, high mitotic rate, etc.) that was confirmed with IHC. They were therefore termed “large cell neuroendocrine carcinomas”. All three were negative for P63, and two of them remained negative for TTF-1.

**Table 2:** Re-classification of 20 Large cell carcinomas of the lung by the staining pattern of P63.

Case	Initial Diagnosis	P63 IHC	TTF-1 IHC	Final Diagnosis
1	LCC	Positive	Positive	SCC
2	LCC	Negative	Positive	AC
3	LCC	Positive	Negative	SCC
4	LCC	Positive	Negative	SCC
5	LCC	Negative	Negative	Neuroendocrine LCC†
6	LCC	Negative	Positive	AC
7	LCC	Positive	Positive	SCC
8	LCC	Positive	Negative	SCC
9	LCC	Negative	Positive	Neuroendocrine LCC†
10	LCC	Positive	Negative	SCC
11	LCC	Negative	Positive	AC
12	LCC	Positive	Negative	SCC
13	LCC	Positive	Negative	SCC
14	LCC	Negative	Positive	AC
15	LCC	Negative	Negative	Neuroendocrine LCC†
16	LCC	Negative	Positive	AC
17	LCC	Negative	Positive	AC
18	LCC	Positive	Negative	SCC
19	LCC	Negative	Positive	AC
20	LCC	Positive	Negative	SCC

† Cases with neuroendocrine differentiation after histological review, confirmed by neuroendocrine IHC markers (synaptophysin and CD56).

### Value of P63 and TTF-1 immunohistochemistry in reducing the “carcinoma not otherwise specified (NOS)” category in small specimens

Results are summarized in Table 3. All P63 positive cases (11/32, 34%) were diagnosed as SCCs (Fig. 2B and 2C) although two of them co-expressed TTF-1. All P63 negative tumors were considered ACs if they showed TTF-1 positivity (13/32, 41%), and only “suggestive of AC” if this latter antibody was not available (5/32, 16%). Finally, in three instances both antibodies were negative (3/32, 9%), and subsequent follow-up was able to identify one adenocarcinoma and one sarcomatoid carcinoma.

**Table 3:** Re-classification of 32 carcinomas (NOS) by the staining pattern of P63 in a prospective series of small thoracic samples.

Case	Type of biopsy	Initial Diagnosis	P63 IHC	TTF-1 IHC	Final Diagnosis
1	Bronchoscopic	Carcinoma	Negative	Negative	Carcinoma^1^
2	Bronchoscopic	Carcinoma	Positive	Negative	SCC^1^
3	Bronchoscopic	Carcinoma	Positive	Negative	SCC^2^
4	Bronchoscopic	Carcinoma	Positive	Negative	SCC
5	Bronchoscopic	Carcinoma	Negative	—	Suggestive of AC
6	Core-needle	Carcinoma	Positive	Negative	SCC^1^
7	Bronchoscopic	Carcinoma	Positive	—	SCC^1^
8	Bronchoscopic	Carcinoma	Positive	Negative	SCC^1^
9	Bronchoscopic	Carcinoma	Negative	—	Suggestive of AC^3^
10	Bronchoscopic	Carcinoma	Positive	—	SCC^1^
11	Bronchoscopic	Carcinoma	Positive	Negative	SCC^1^
12	Bronchoscopic	Carcinoma	Negative	—	Suggestive of AC
13	Bronchoscopic	Carcinoma	Negative	Positive	AC
14	Core-needle	Carcinoma	Negative	Positive	AC^4^
15	Bronchoscopic	Carcinoma	Negative	Positive	AC
16	Bronchoscopic	Carcinoma	Negative	Positive	AC^5^
17	Bronchoscopic	Carcinoma	Negative	—	Suggestive of AC
18	Bronchoscopic	Carcinoma	Negative	—	Suggestive of AC
19	Bronchoscopic	Carcinoma	Positive	Positive	SCC
20	Core-needle	Carcinoma	Negative	Positive	AC
21	Bronchoscopic	Carcinoma	Negative	Positive	AC^3^
22	Core-needle	Carcinoma	Negative	Positive	AC
23	Bronchoscopic	Carcinoma	Positive	—	SCC
24	Bronchoscopic	Carcinoma	Positive	Positive	SCC^1^
25	Bronchoscopic	Carcinoma	Negative	Positive	AC^4^
26	Bronchoscopic	Carcinoma	Negative	Negative	Carcinoma^6^
27	Bronchoscopic	Carcinoma	Negative	Positive	AC
28	Bronchoscopic	Carcinoma	Negative	Negative	Carcinoma^7^
29	Bronchoscopic	Carcinoma	Negative	Positive	AC
30	Core-needle	Carcinoma	Negative	Positive	AC
31	Bronchoscopic	Carcinoma	Negative	Positive	AC^4^
32	Bronchoscopic	Carcinoma	Negative	Positive	AC^4^

1 *KRAS* and *EGFR* wild type tumour;

2 Confirmed after surgical excision;

3 Tumour with *EGFR* gene amplification;

4 *KRAS* mutant tumours (G12C or G12V);

5 *EGFR* mutant tumour (E746-A750del);

6 Sarcomatoid carcinoma confirmed after surgical excision;

7 AC confirmed in a subsequent pleural effusion.

## Discussion

We have shown the clinical utility of P63 IHC for the identification of lung SCCs, further validating our previous microarray study. That P63 is a marker of squamous differentiation is well known and overexpression of this gene has been consistently identified in lung SCCs by global gene expression profiling or by IHC [14]–[20]. The reported positivity by this latter method is usually over 80% in most series, but it should be emphasized that better differentiated areas and even well-differentiated tumors may be negative [10], [12], [18], [21], [22]. This fact may explain the comparatively low rate of positivity in our two validation series (Fig. 1 and Table 1) using TMAs (76% and 54%). Fortunately, this is not a problem in clinical samples because IHC is not needed in well differentiated SCC. Nonetheless, the specificity of P63 IHC has been challenged. Although from 0% to 33% of lung ACs may express P63, negative P63 IHC is used when researchers need to accurately identify ACs for other purposes [9], [12], [21], [23]–[26]. These differences maybe explained by variability at two phases of the procedure: (1) the antibody that has been used to detect P63 (analytical phase), and (2) the interpretation (post-analytical phase) of the staining. The first possibility is less likely [27]. Although ΔNp63 isoforms are frequently expressed in SCCs [28], most of the IHC studies of P63 expression use antibodies that detect all P63 isoforms (TAp63α, TAp63β, TAp63γ, ΔNp63α, ΔNp63β, ΔNp63γ) [10], [27], [29], [30]. In agreement with other authors, we believe that, from a practical point of view, faint or focal immunostaining for P63 should be considered non-specific until there is proof that it is not [10]. Therefore, to increase the specificity of P63 IHC, we considered a positive result when high intensity staining was present in ≥50% of tumor cells [10]. Accordingly, some authors have demonstrated that when using this approach, fewer ACs are P63 positive [31]. Ang et al. have recently reported that P63 maybe positive (>20% tumor cells) or focal (≤20% tumor cells) in 6% and 23% of ACs, respectively, whereas this tumor type exhibits very rarely (1.6%) diffuse staining (>50% tumor cells) [31].

Along these lines, several other approaches have been proposed to improve the classification of lung carcinomas. Such procedures include the use of a combination of markers (CD63, P63 and CD56 or TTF-1, CK 5/6, and P63 or a five-antibody test comprising TRIM29, CEACAM5, SLC7A5, MUC1, and CK5/6), the use of novel antibodies (democollin-3) or even microRNA expression [12], [26], [30], [32], [33]. Interestingly, the desmocollin-3 proposal was in fact derived from our microarray study (page 710 in reference 30), because this was indeed the top differentially expressed gene [3]. We chose to validate P63, in spite of its lower fold-change, because of the reproducibility of a nuclear staining and the availability of the antibody (i.e. P63 IHC is routinely used for assessing the *in situ* versus infiltrative nature of breast and prostate carcinomas) [34]–[36]. Overall, the methodologies taken by other researchers to raise specificity may also lower the likelihood of clinical application because of the very limited material that is usually obtained in bronchoscopic or core-needle biopsies. Interestingly, another group has recently arrived at similar conclusions although their specific data is not shown [37].

After we had validated our microarray data in two independent series, we wanted to address two of the clinically relevant problems in lung targeted therapies. Both surgically resected and unresectable biopsy-proven lung carcinomas with a non-specific diagnosis (i.e., termed “LCC” in the former case and “carcinoma NOS” in the latter) may eventually be considered for a targeted therapy that must exclude SCCs. Assuming, based on our previous evidence, that P63 positive cases are *bona fide* SCCs, we were able to demonstrate the usefulness of P63 IHC in a series of surgically resected LCCs and in a prospective cohort of small specimens. One could argue that there is no “gold standard” in these two situations, which is true, but this approach parallels the real clinical work. The term “LCC” is defined as one of exclusion and, as such, this category has been questioned. Indeed, in microarray experiments these cases belong to either the AC or the SCC group [20], [38]. Therefore, the diagnosis of LCCs is not reproducible and depends on several uncontrollable parameters (sampling, expertise, etc.). On the other hand, in the real clinical world, we are constantly asked to refine the “carcinoma NOS” group in order to guide the oncologist's therapeutic decision. In our setting, in over 70% of the biopsies of the unresectable lung carcinomas, neither keratin nor gland formation were identified.

In summary, we have demonstrated how the use of P63 IHC with rigid interpretation criteria can effectively improve the identification of SCCs. Targeted therapies in the field of lung cancer need more reproducible histological diagnoses.
